# Gut Microbiota Alter Colonic Expression of Genes Encoding Drug Transporters and Drug-Metabolizing Enzymes in Mice

**DOI:** 10.3390/ijms27177583

**Published:** 2026-08-24

**Authors:** Douglas A. Nelson, Vaishnavi Veerareddy, Xiaojia Tang, Purna C. Kashyap, Krishna R. Kalari, Karunya K. Kandimalla

**Affiliations:** 1 Department of Pharmaceutics, Brain Barriers Research Center, College of Pharmacy, University of Minnesota, Minneapolis, MN 55455, USA; 2 Department of Quantitative Health Sciences, Mayo Clinic, Rochester, MN 55905, USA; 3 Division of Gastroenterology and Hepatology, Department of Medicine, Mayo Clinic, Rochester, MN 55905, USA

**Keywords:** gut microbiota, drug metabolism, drug transport

## Abstract

Gut microbiota regulate the intestinal expression of drug-metabolizing enzymes, transporters, and barrier properties that determine oral drug absorption and disposition. This study compares germ-free Swiss Webster mice to mice colonized with fecal microbiota from a single human donor, evaluating microbiota-driven differences in colonic gene expression and P-gp protein expression, as well as small intestinal mucosal permeability. Transcriptomic analysis of colonic tissue revealed microbiota-induced upregulation of genes encoding P-gp and other drug transporters, including MCT1 and OCTN2. Immunofluorescence also indicated greater P-gp expression and apical localization in colonized mice. Gene expression of drug-metabolizing enzymes was also higher in colonized mice, including phase I enzymes (carboxylesterase 2 paralogs), phase II enzymes (UDP-glucuronosyltransferases and glutathione S-transferases), and enzymes responsible for synthesizing their co-substrates, UDP-glucuronic acid and glutathione. Small intestinal mucosal explants demonstrated lower permeability to ^14^C-labeled polyethylene glycol 4000 in colonized mice compared to germ-free mice. In the colon, transcriptomic analysis identified microbiota-dependent changes in genes regulating paracellular tight junctions and actomyosin contractility. These results show that human-derived gut microbiota alter gene expression in the mouse colon, identifying candidate microbiota-responsive genes relevant to oral drug absorption and disposition.

## 1. Introduction

The human gut microbiome influences drug bioavailability through multiple mechanisms. In addition to directly supplying drug-metabolizing enzymes, gut bacteria regulate the expression of intestinal enzymes, transporters, tight junctions, and other molecules affecting oral drug absorption and disposition.

The extent to which intestinal transporters and enzymes affect oral drug disposition depends in large part on their permeability and solubility [1]. High-permeability, high-solubility drugs, such as acetaminophen, pass rapidly through the intestinal epithelium, largely bypassing transporters and metabolizing enzymes [2]. Drugs with low permeability and high solubility, such as metformin, often depend on transporter-mediated uptake for absorption [3]. Substrates of P-glycoprotein (P-gp) with low to moderate permeability, such as fexofenadine, are especially sensitive to P-gp-mediated efflux, resulting in limited intestinal absorption [4,5]. Highly lipophilic compounds like simvastatin [6,7] or cyclosporine [8] tend to partition into enterocyte membranes, making them particularly susceptible to metabolism by membrane-bound cytochrome P450 enzymes [9]. Finally, while the vast majority of orally absorbed drugs cross the intestinal epithelium by transcellular pathways [10], hydrophilic compounds like lactulose [11] are absorbed paracellularly through tight junctions [12].

Two primary enzymatic pathways that drive drug metabolism are considered in this paper: phase I metabolism, which modifies drugs via oxidation (primarily by cytochrome P450 enzymes), reduction, or hydrolysis; and phase II metabolism, which forms drug metabolites by conjugation to increase their solubility and facilitate excretion. While phase II metabolism often follows phase I, some drugs undergo only one of these processes [13].

Microbiota density increases by roughly ten orders of magnitude from the proximal small intestine to the colon, which implies a progressively stronger microbial influence [14]. This variation affects both direct microbial metabolism of drugs and microbiota-mediated regulation of intestinal enzymes, transporters, and tight junctional integrity [15].

The small intestine is the primary site of drug absorption, with the duodenum, jejunum, and ileum accounting for most systemically absorbed drugs [16]. The relative contribution of each small intestinal segment varies by drug. For example, drugs with high solubility and high permeability can be largely absorbed in the duodenum before reaching other sections. In contrast, drugs with lower solubility tend to be absorbed more efficiently in the jejunum and ileum due to their extensive microvillar surface area [17]. Despite the primacy of the small intestine, the colon remains relevant for formulations targeting local conditions such as inflammatory bowel disease. In addition, some systemically active drugs, such as acetylsalicylic acid, exhibit appreciable colonic absorption [18,19]. Although absorption in the colon is generally limited, systemically delivered drugs can also reach the colon through biliary excretion. For example, the chemotherapeutic irinotecan is detoxified by glucuronide conjugation in the liver, then excreted into the gut, where it may be reactivated by bacterial β-glucuronidases, leading to nonspecific toxicity in healthy colon tissue [20].

This study examines microbiota-induced changes in gene expression in the colon, where regulation of transporters, enzymes, and barrier properties is expected to be strongest. The findings are hypothesis-generating for intestinal drug absorption and disposition, with greater uncertainty in more proximal tissues. The study also directly assesses the effect of microbiota on small intestinal barrier function.

## 2. Results

### 2.1. Transcriptomic Overview and Differential Gene Expression

**Figure 1:**
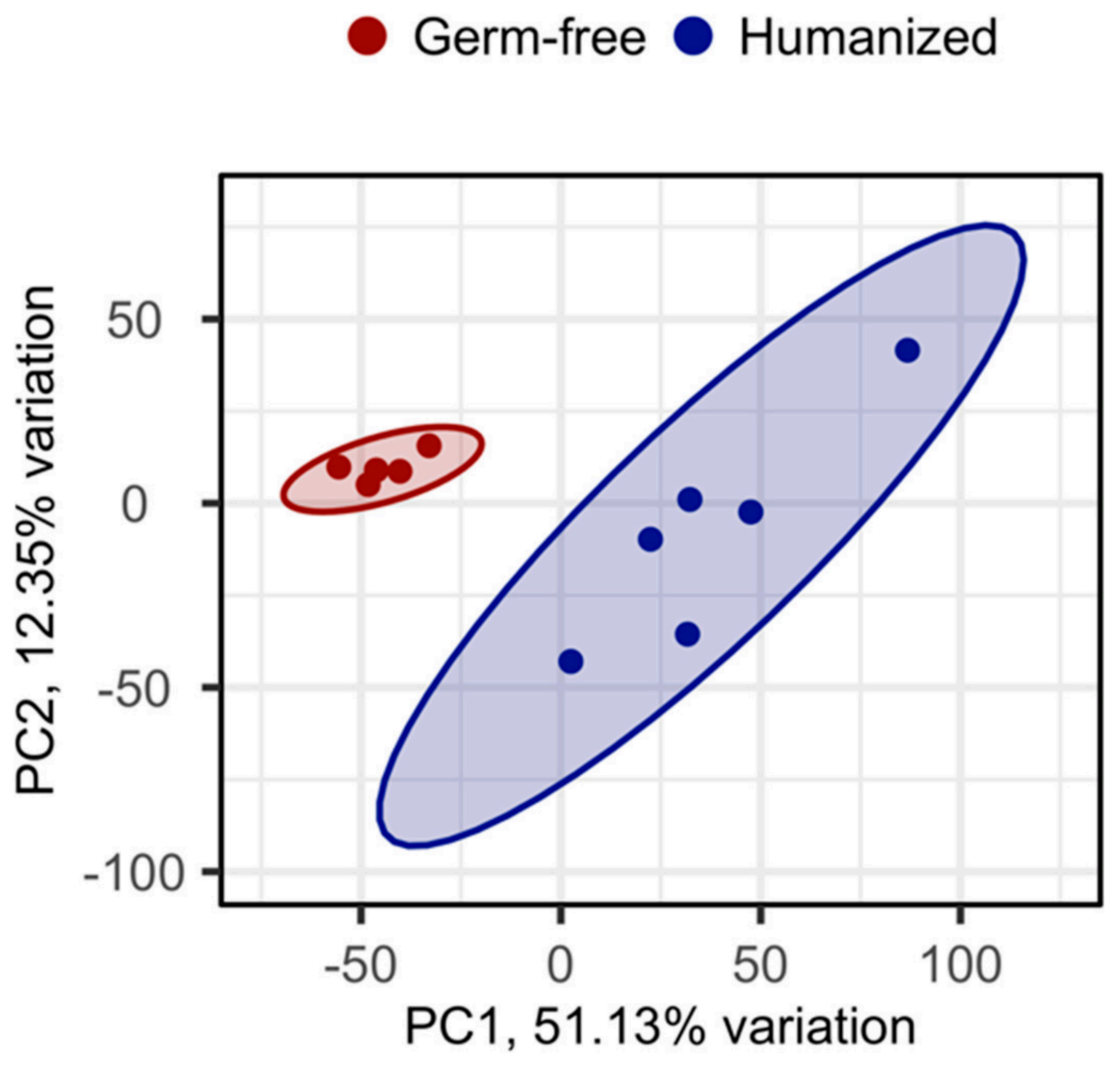
PCA of RNA-Seq results for colons of five germ-free (red) and six humanized (blue) mice. Ellipses represent 95% multivariate t-distribution data ellipses for each group.

The principal component analysis (PCA) plot (Figure 1) illustrates that germ-free and humanized mice represent distinct transcriptomic profiles. These groups form clusters well-separated by the first principal component (PC1), which represents 51% of the overall gene-wise variance and largely accounts for the variance among germ-free samples. Both principal components contribute to variation among humanized mice, which show higher overall dispersion than germ-free mice. A comparison of the expression profiles of 19,863 genes in humanized mouse colon to germ-free mouse colon resulted in 3826 differentially expressed genes, defined as having corrected *p*-values (FDR) of less than 0.01. The number of differentially expressed genes at various thresholds of statistical significance and magnitude is presented in Table 1.

### 2.2. Immune Response and Extracellular Matrix

**Figure 2:**
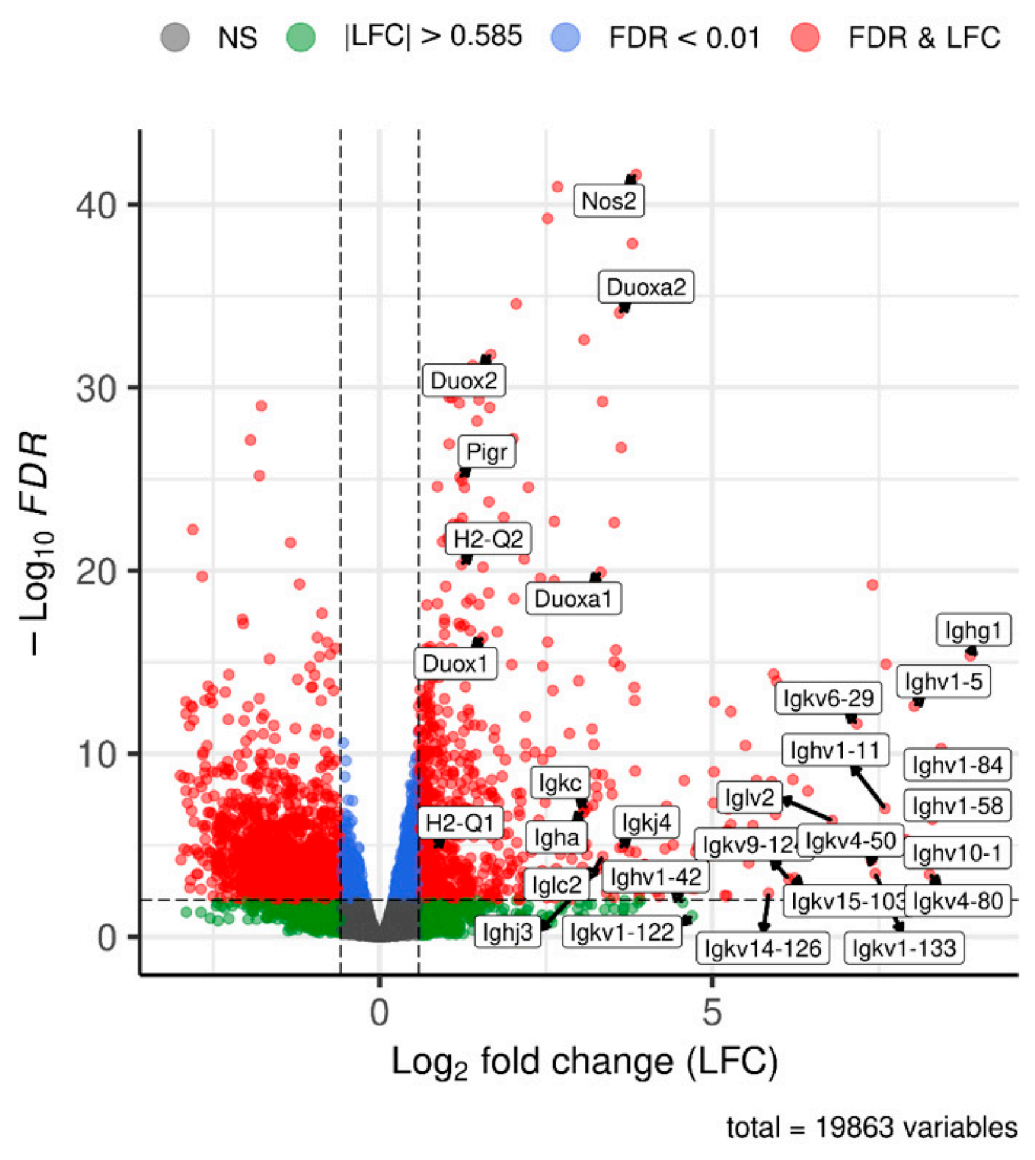
Volcano plot shows upregulation of genes related to innate and adaptive immunity in mice with humanized gut microbiota. As with Table 1, blue denotes genes with FDR <0.01, green represents genes with absolute log_2_-fold change > 0.58 (corresponding to fold changes of 2/3 and 3/2), and red indicates genes with both FDR < 0.01 and absolute log2-fold changes >0.58.

The introduction of human microbiota resulted in a general increase in gene expression related to adaptive immunity, including constant and variable immunoglobulin chains comprising IgG and IgA, as well as the polymeric immunoglobulin receptor (*Pigr*), crucial for epithelial secretion of immunoglobulins. Although immunoglobulin chains exhibited the largest mean fold changes, *p*-values were relatively high because of high sample dispersion. Genes central to innate immunity were moderately upregulated with relatively uniform inter-sample expression and high statistical significance. These include the reactive oxygen species (ROS)-producing nitric oxide synthase (*Nos2*) and dual oxidase complex genes (*Duox1*, *Duox2*, *Duoxa1*, *Duoxa2*). Log_2_-fold changes and FDR *p*-values illustrating this immune response are given in Figure 2.

**Figure 3:**
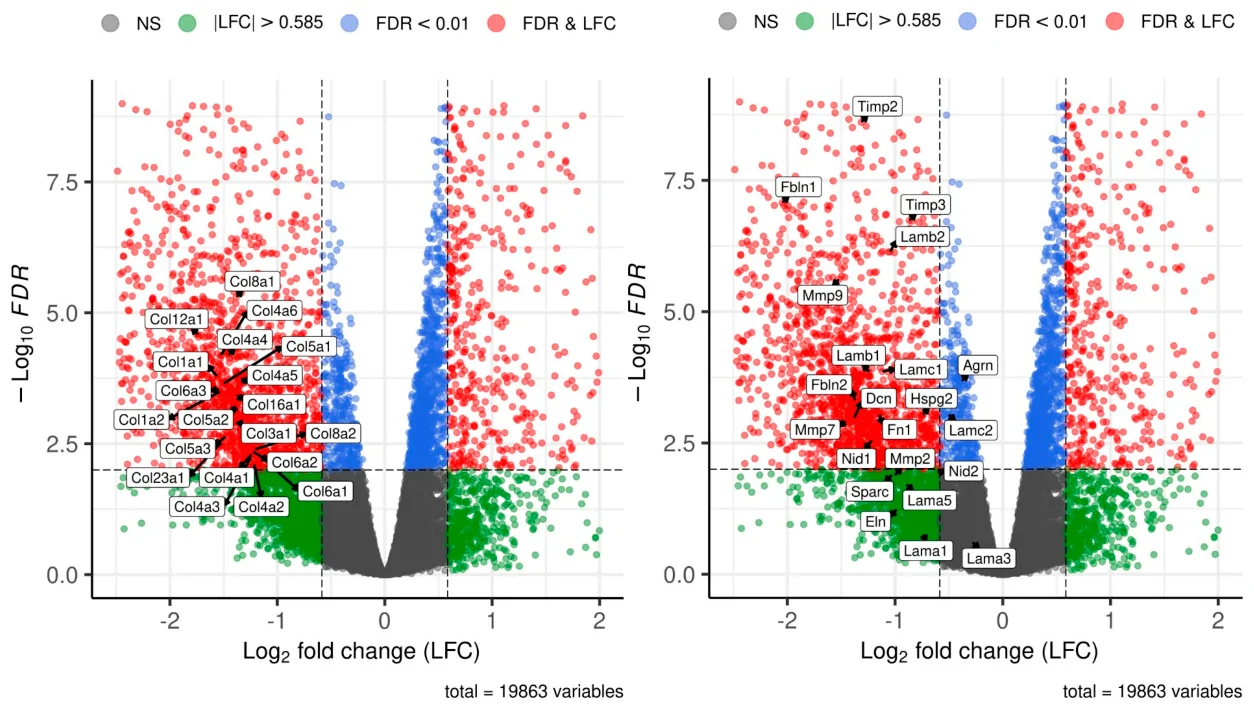
Genes encoding all the major extracellular matrix components were downregulated in humanized mouse colon compared to those of germ-free mice. Additionally, colons from humanized mice also showed lower expression of genes related to ECM turnover.

Genes encoding all major extracellular matrix (ECM) constituents and regulators of ECM remodeling were uniformly downregulated in humanized mouse colon (Figure 3). This includes genes encoding collagens in the basement membrane (collagen IV, VI, VIII, and XXIII) and the interstitial matrix (collagens I, III, V, XXII, and XVIII) [21]. Other ECM protein-coding genes exhibiting similar differential expression included several key intestinal laminin subunits, as well as fibulins (*Fbln1*, *Fbln2*), nidogens (*Nid1*, *Nid2*), fibronectin (*Fn1*), decorin (*Dcn*), perlecan (*Hspg2*), agrin (*Agrn*), and osteonectin (*Sparc*). Additionally, humanized mouse colon also exhibited lower expression of genes involved in ECM turnover, including metalloproteases (*Mmp7*, *Mmp9*, *Mmp2*) and metalloprotease inhibitors (*Timp2*, *Timp3*).

**Figure 4:**
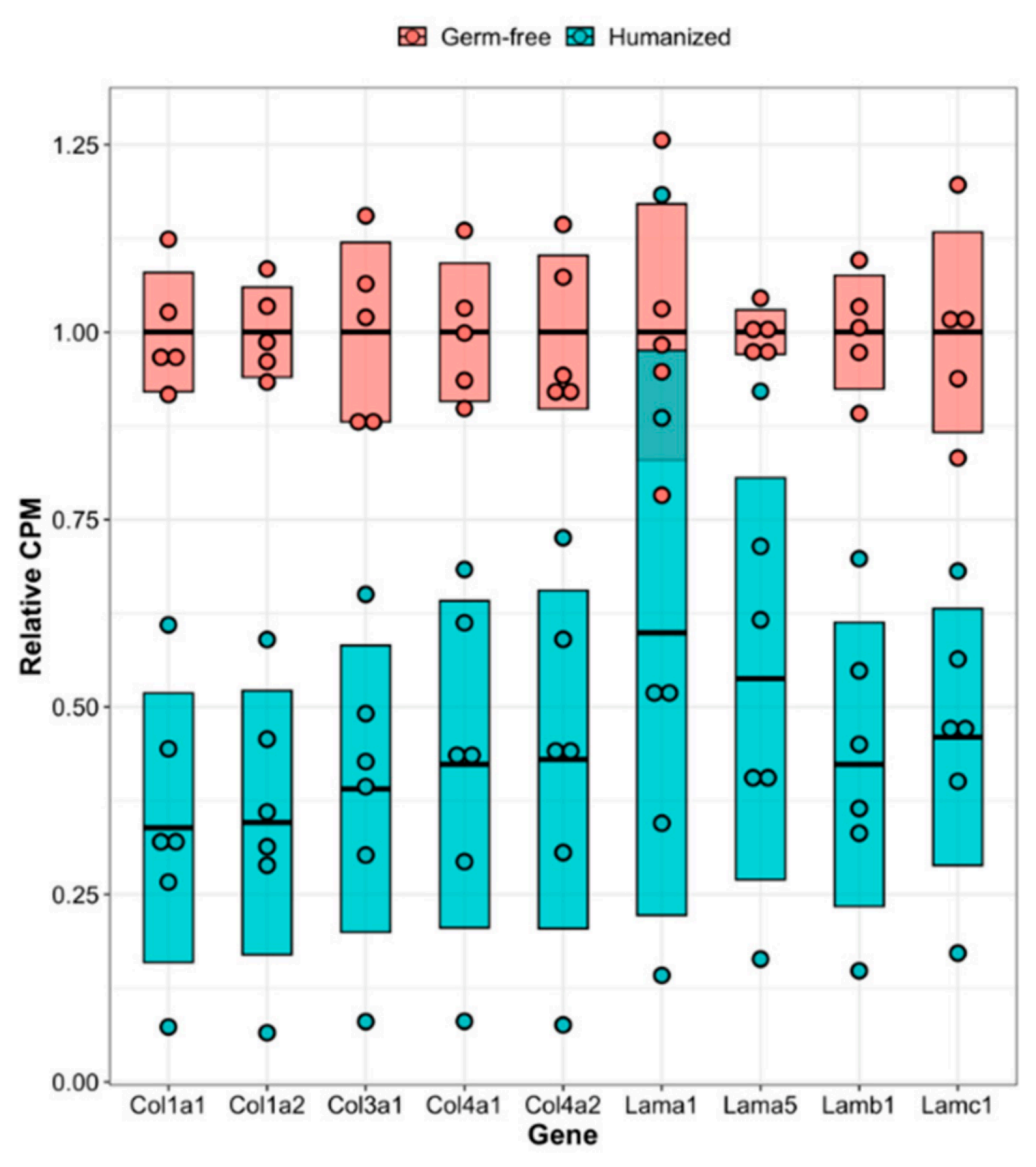
mRNA expression for key extracellular matrix genes normalized to germ-free means. Included are genes encoding the predominant proteins comprising the basement membrane and interstitial matrix. The plot shows greater absolute dispersion among humanized mice.

Despite being downregulated, collagen and laminin genes show higher inter-sample variability in humanized samples. This dispersion is illustrated in Figure 4, which includes the genes encoding the predominant collagen and laminin isoforms (collagen IV, laminin-111, and laminin-511 of the basement membrane, as well as collagen I and collagen III of the interstitial matrix).

### 2.3. Tight Junctions and Water Channels

Table 2 presents the expression analysis of genes encoding proteins involved in tight junction structure (claudins, occludin, and zonula occludens proteins) and actomyosin contractility (myosin II and myosin light chain kinase). Zonula occludens-3 (*Tjp3*), claudin-23 (*Cldn23*), and claudin-4 (*Cldn4*) were upregulated in humanized mouse samples. All upregulated tight junction genes were highly expressed in the top RPKM quartile, with *Tjp3* being the most abundant zonula occludens family member. At the same time, myosin light chain kinase (*Mylk*), the myosin II regulatory light chain (*Myl9*), and the smooth muscle myosin heavy chain (*Myh11*) were downregulated.

Table 3 lists four aquaporin genes with biologically relevant expression. Most notably, the highly abundant water channel aquaporin 8 (*Aqp8*) is upregulated 375-fold.

### 2.4. Permeability

**Figure 5:**
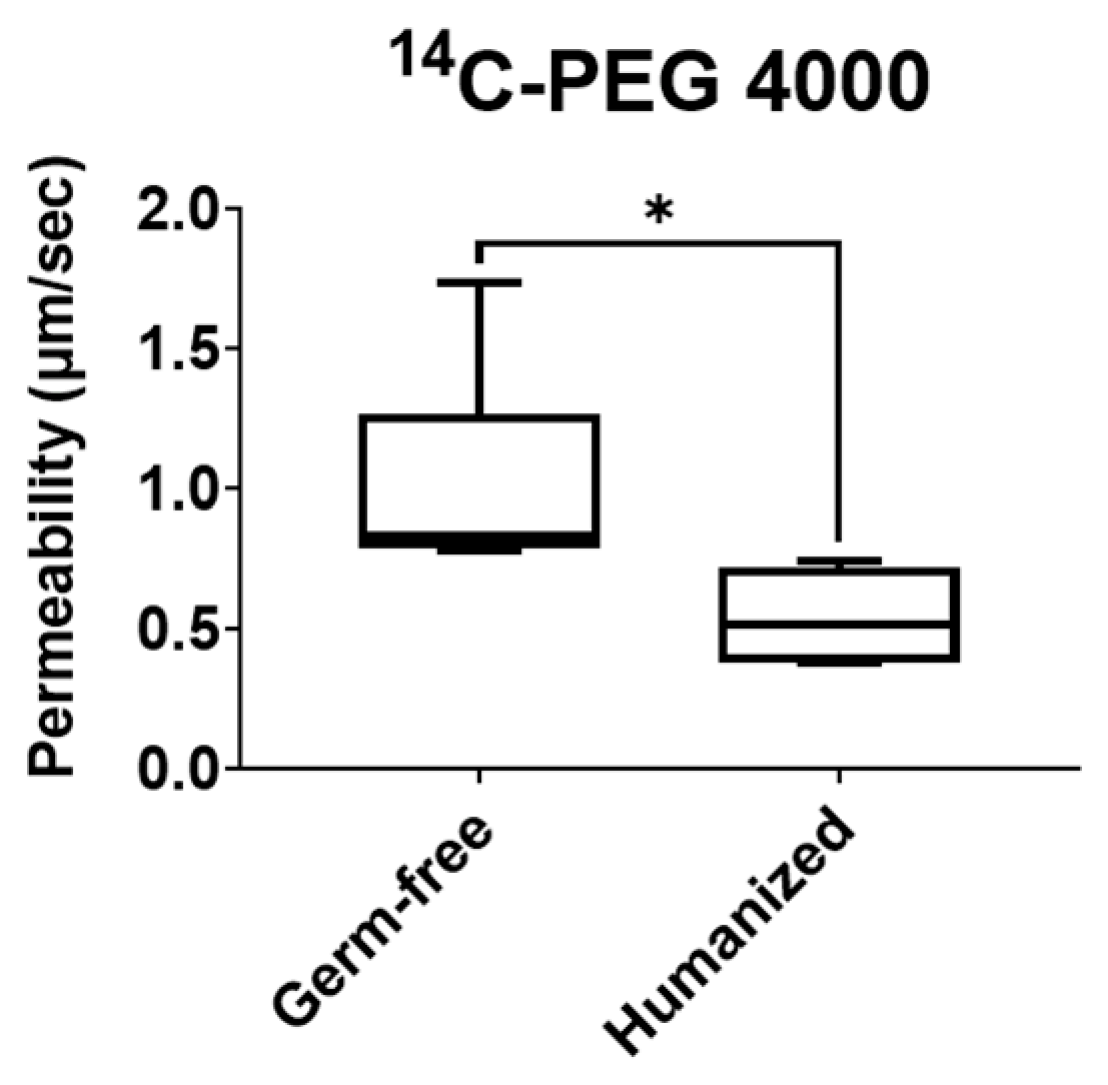
Ussing chamber experiments measuring flux of ^14^C-PEG 4000 across small intestinal tissue from germ-free and humanized mice. Two-tailed Welch’s *t*-test. * *p*-value < 0.05.

Ussing chamber experiments measuring ^14^C-PEG 4000 flux across small intestinal tissue from germ-free and humanized mice were used to measure intestinal permeability. A two-tailed Welch’s *t*-test was used to compare humanized and germ-free samples (Figure 5). Results suggest that permeability was higher in germ-free mice compared to humanized mice (mean difference = 4.75 × 10^−5^ μm/s, 95% CI: 6.12 × 10^−6^ to 8.89 × 10^−5^, *p* = 0.0296, Hedges’ g = 1.36).

### 2.5. Phase I Drug-Metabolizing Enzymes

**Figure 6:**
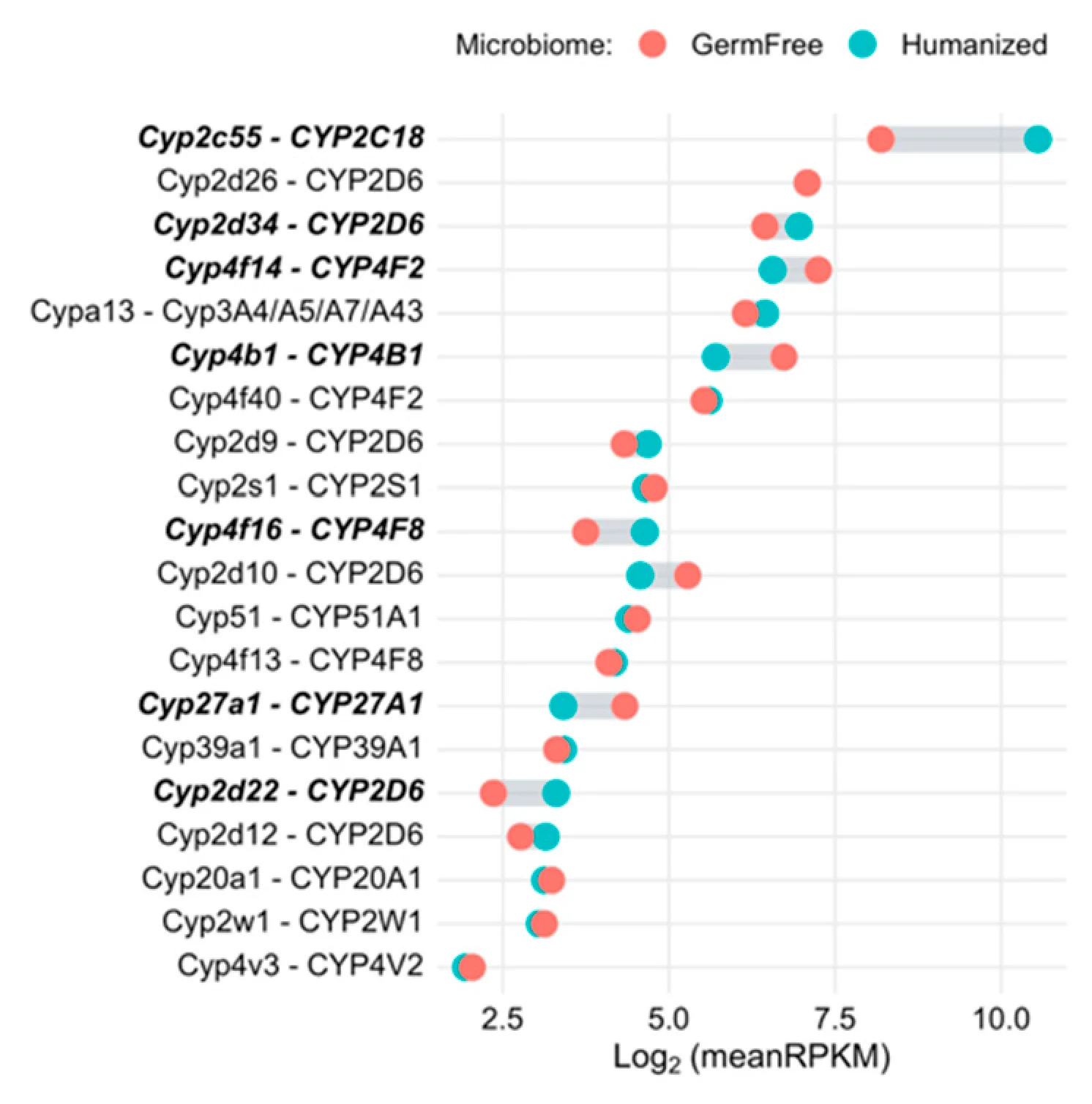
List of cytochrome P450 genes with human homologs and log_2_(RPKM) > 2 (RPKM > 4). Mus musculus gene names are given, followed by human homologs. Genes in bold are differentially expressed with FDR < 0.01. None of the differentially expressed genes are homologs of human enzymes with known relevance for intestinal drug metabolism.

Relative mean expression values for cytochrome P450 genes in germ-free and humanized mouse colons are shown in Figure 6. Out of 20 genes, four are upregulated, and three are downregulated. None of these, however, correspond to important human drug-metabolizing enzymes. At the same time, NADPH–cytochrome P450 reductase (*Por*), the essential electron donor for all cytochrome P450 activity, was upregulated in humanized colon by 40% (FDR = 9 × 10^−7^).

Relative abundance and differential expression results for hydrolases and reductases involved in phase I metabolism are provided in Table 4. All the mouse homologs of the human gene *CES2* were upregulated in humanized mice, including *Ces2a*, which increased 24-fold with FDR = 1.3 × 10^−183^. In addition, two carbonyl reductase genes (*Cbr1* and *Cbr3*), *Akr1b8*, a homolog of the human aldo-keto reductase gene *AKR1B10*, and the quinone reductase gene *Nqo1* were also upregulated.

### 2.6. Phase II Drug-Metabolizing Enzymes

Five phase II enzyme groups that catalyze acetylation, glucuronidation, glutathione S-conjugation, methylation, and sulfoconjugation were tested for over-representation of differentially expressed genes in phase II enzyme groups (Table 5). No genes encoding phase II enzymes were downregulated, while genes encoding UDP-glucuronosyltransferases (UGTs), glutathione S-transferases (GSTs), and microsomal glutathione S-transferases (MGSTs) were over-represented among the upregulated genes. In addition, all five genes encoding enzymes that synthesize the corresponding co-substrates UDP-glucuronic acid and glutathione were upregulated (Table 6).

### 2.7. Transport: ATP-Binding Cassettes and Solute Carriers

**Figure 7:**
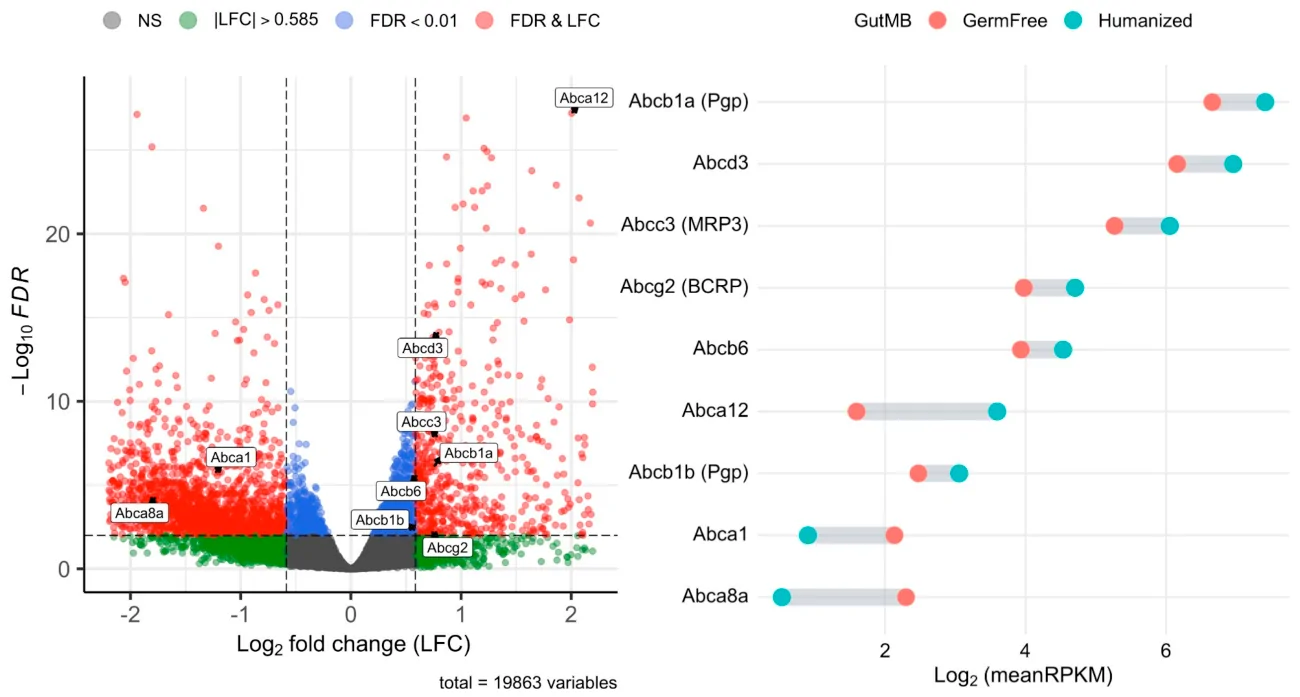
**Left**, volcano plot of differentially expressed ATP-binding cassette genes with RPKM > 4 (log_2_(RPKM) > 2). **Right**, relative abundance of the same genes, indicated by log_2_(RPKM). Both mouse homologs of the human gene *ABCB1*, which encodes the major drug efflux transporter P-gp, were upregulated. Also upregulated were *Abcc3*, which encodes MRP3, and *Abcg2*, the mouse ortholog of human *ABCG2*, which encodes BCRP. *Abcg3*, another BCRP-related mouse gene, had very low expression and was not differentially expressed. In humans, *ABCA12* is primarily expressed in skin. *Abcb6* and *Abcd3* encode proteins involved in iron and fatty acid transport, respectively. Because of their lack of relevance, *Abca12*, *Abcb6* and *Abcd3* are not discussed further.

The seven most abundant differentially expressed ATP-binding cassette transporters were upregulated in humanized mice, as shown in Figure 7. These include both mouse homologs of the human gene *ABCB1*, which codes for P-gp: *Abcb1a* (LFC = 0.75, FDR = 8 × 10^−7^) and *Abcb1b* (LFC = 0.58, FDR = 5 × 10^−3^). Also upregulated were *Abcc3*, which encodes multidrug resistance-associated protein 3 (MRP3), and *Abcg2*, the mouse ortholog of human breast cancer resistance protein (BCRP).

**Figure 8:**
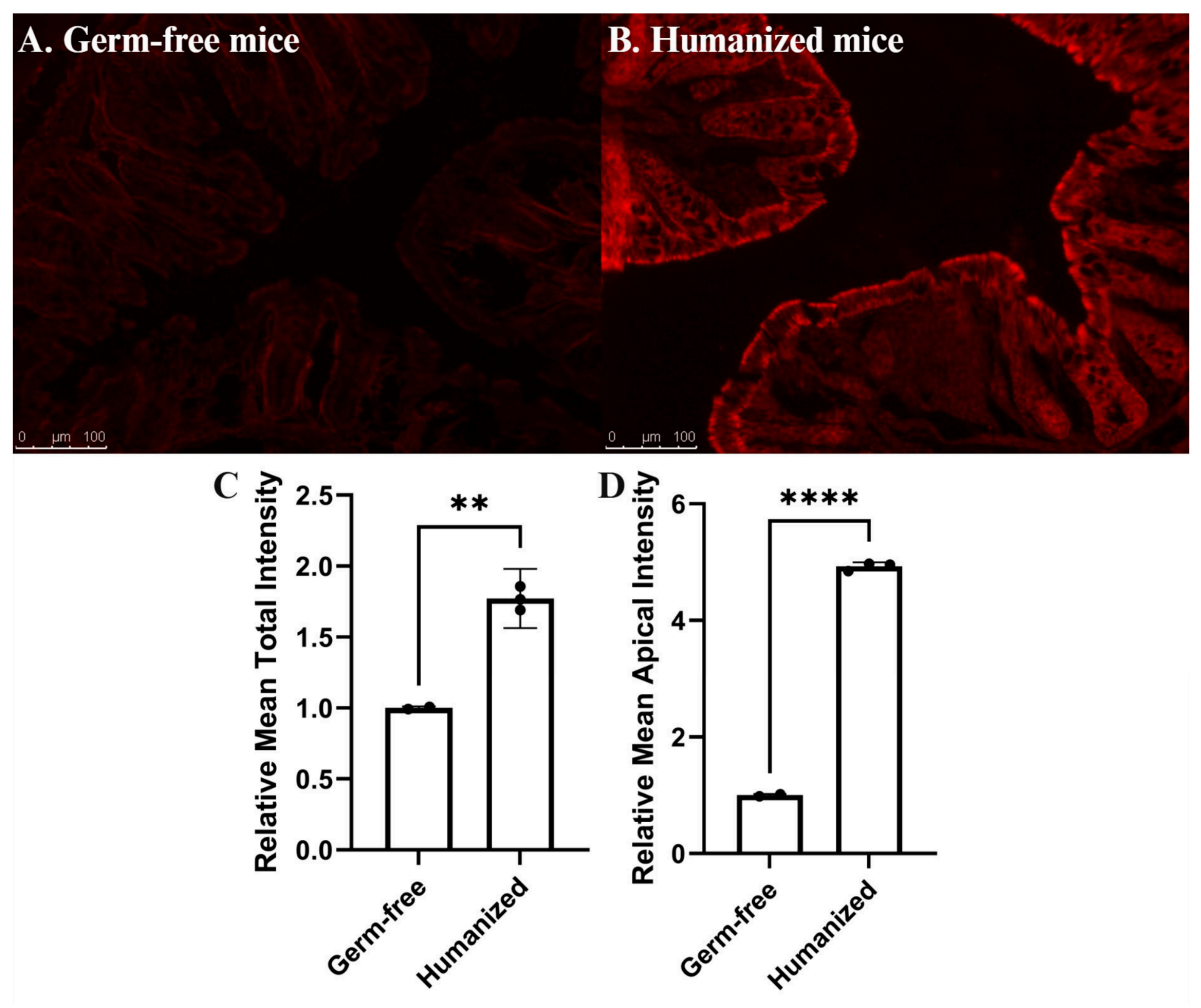
Higher P-gp protein expression in the colon of humanized mice compared to germ-free mice is shown by immunofluorescence. Colonic sections from germ-free (**A**) and humanized (**B**) mice were stained for P-gp and tagged with Cy3-conjugated streptavidin dye. Relative mean intensity for two germ-free and three humanized mice, with 95% confidence intervals, is shown in (**C**), and mean intensity along the apical surface is shown in (**D**). Scale bar: 100 μm. Fold changes were 1.8 for mean intensity (**C**) and 5.0 for apical surface mean intensity from 50 manually selected points (**D**). By an unpaired two-tailed *t*-test, *p*-values were 0.0012 and <0.0001 for total and apical surface differences, respectively. (** *p* < 0.01, **** *p* < 0.0001).

An observed increase in P-gp protein expression by immunofluorescence, shown in Figure 8, was concordant with the transcriptomic result. Mean intensity after background removal was 77% higher in humanized mice. The nominal *p*-value for the immunofluorescence comparison was 0.0012. Although the analysis was based on only *n* = 2 germ-free and *n* = 3 humanized mice, limiting the reliability of the statistical comparison, the localization of P-gp on the apical surface was markedly higher in humanized mice than in the germ-free colon.

Out of 306 solute carrier genes, 101 were differentially expressed. Table 7 lists differentially expressed solute carriers with known drug substrates. Notably, the major apical intestinal influx drug transporters—monocarboxylate transporter 1 (MCT1), encoded by *Slc16a1*, and organic cation transporter novel family member 2 (OCTN2), encoded by *Slc22a5*—were upregulated.

### 2.8. Colonoid Efflux Studies

**Figure 9:**
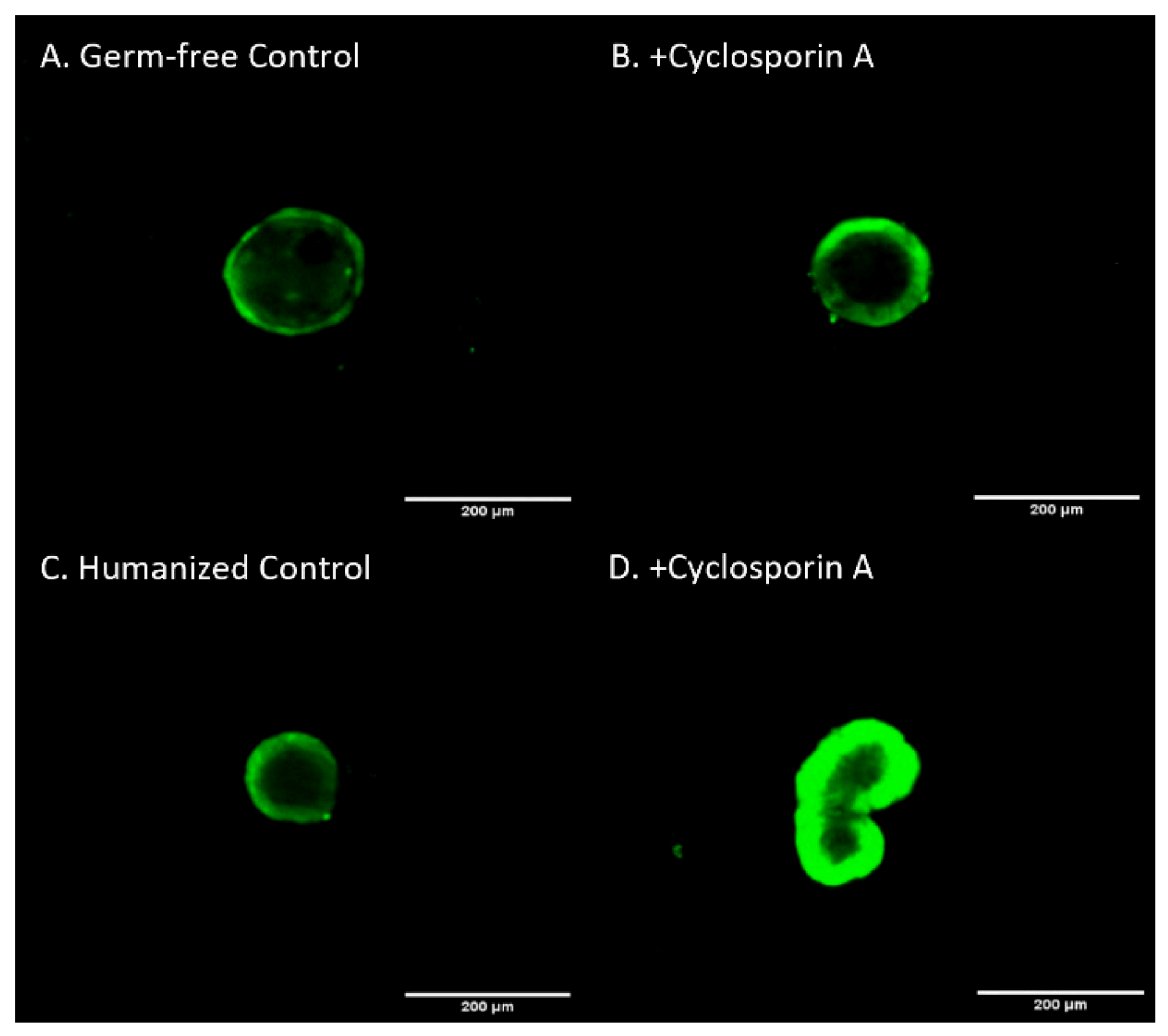
Calcein-AM fluorescence imaging in colonoids: Colonoids from germ-free and humanized mice were pretreated with cyclosporine A, a P-gp inhibitor, for 30 min, followed by treatment with calcein-AM for 60 min at 37 °C. Confocal images show calcein-AM accumulation in germ-free colonoid control (**A**) and cyclosporine A pretreated colonoid (**B**), along with control from humanized mice (**C**) and cyclosporine A pretreated colonoid (**D**). Scale bar: 200 μm.

Live imaging of colonoids incubated with calcein-AM revealed differences in fluorescence intensity between experimental groups. In colonoids from both germ-free and humanized colons, cyclosporine A pretreatment increased intracellular fluorescence compared to untreated controls, particularly along the apical surface, suggesting reduced calcein-AM efflux by P-gp and/or greater intracellular conversion of calcein-AM by esterases. In addition to apical fluorescence differences with and without inhibitor treatment, colonoids derived from humanized mice exhibited higher fluorescence intensity than those from germ-free mice. Representative images for each condition are shown in Figure 9.

## 3. Discussion

Microbial colonization produced extensive changes in host gene expression. Using a strict 1% false discovery rate threshold, we identified approximately 4000 microbiota-sensitive genes (Table 1). These included genes involved in drug disposition, such as active transporters and drug-metabolizing enzymes, as well as genes related to epithelial barrier regulation, immune responses, ECM turnover, and water transport.

Colonization establishes a dynamic host–microbiota system characterized by bidirectional interactions between microbial composition and host adaptive immune responses [25,26]. As expected, both adaptive immune and innate immune genes were uniformly upregulated in response to colonization (Figure 2). The latter is consistent with prior studies showing specifically that lipopolysaccharides from Gram-negative bacteria can induce expression of *Nos2* and *Duox2* [27,28]. While these immune changes have limited direct relevance for small-molecule oral drug disposition, and biologics are not usually administered orally, microbiota-driven changes in intestinal immunity may propagate to other drug-relevant mucosal interfaces through the common mucosal immune system [29,30].

Genes encoding basement membrane constituents and regulators of ECM turnover were uniformly downregulated in humanized mouse colon relative to germ-free mice (Figure 3), suggesting microbiota modulate ECM homeostasis. While the basement membrane is not generally a rate-limiting barrier to the transport of small-molecule drugs, it regulates epithelial barrier integrity through ligand–receptor signaling and mechanotransduction [31,32].

Ussing chamber permeability of ^14^C-PEG 4000 through the small intestinal mucosal explant appeared higher in germ-free mice than in mice with human microbiota (Figure 5). Although connecting the colonic gene-expression findings to the small intestinal permeability result would require confirmation in small intestinal tissue and mechanistic studies, the hypotheses they suggest are worth consideration: microbial colonization increased colonic expression of relevant claudins while downregulating myosin contractility machinery (Table 2), a relatively novel and often overlooked potential contributor to reduced paracellular permeability.

Two distinct pathways mediate tight junction permeability: the pore pathway, a high-throughput route with high charge and size selectivity, and the leak pathway, a low-capacity, nonselective route that can accommodate larger solutes such as PEG 4000. The leak pathway is primarily regulated by zonula occludens and occludin, while certain claudin proteins form selective pores [33].

*Cldn4* was upregulated in humanized mouse colon (Table 2). Claudin-4 is a tight junction protein whose increased expression has been associated with reduced paracellular permeability and increased epithelial barrier resistance [34,35]. Reduced expression of Mylk, which encodes myosin light-chain kinase (MLCK), and Myl9, which encodes a myosin regulatory light chain phosphorylated by MLCK, was also observed. MLCK-dependent phosphorylation of myosin light chain promotes actomyosin contraction and increases epithelial permeability [36,37]. Reduced *Mylk* expression has similarly been shown to reduce tight junction permeability in intestinal epithelial monolayers [38]. In addition to *Cldn4*, the less canonical *Cldn23* was also upregulated. Recent studies have shown that together Claudin-4 and Claudin-23 proteins can form highly size- and charge-selective paracellular channels and, in some configurations, may form poreless junctions [39].

Among the most dramatic changes induced by microbiota is the 375-fold upregulation of the major colonic water channel encoded by *Aqp8* (Table 3) [40]. While the mechanism underlying this remarkable increase remains unclear, increased *Aqp8* expression could alter luminal water volume and potentially affect the dissolution of poorly soluble compounds.

Among the differentially expressed cytochrome P450 genes (Figure 6), none are relevant homologs for human intestinal drug metabolism [41,42]. However, little can be inferred from this result given the substantial divergence between mouse and human cytochrome P450 systems, including the larger and distinct set of mouse isoforms [43]. This point is illustrated by the use of pharmacokinetic mouse models that replace 33 cytochrome P450 mouse genes with six key human enzymes [44,45].

Several non-cytochrome phase I hydrolases and reductases were substantially upregulated in humanized mice (Table 4). Most notably, all four mouse homologs of the human gene *CES2* were upregulated, with *Ces2a* increasing 24-fold (FDR = 10^−183^). These four mouse paralogs map to a single human gene, CES2, the major intestinal hydrolase responsible for metabolizing drugs like clopidogrel and prodrugs like oseltamivir [46,47], although the individual roles of the mouse paralogs remain incompletely understood [48]. *Ces1g* was also upregulated, though expression of the human gene *CES1* is predominantly hepatic with limited intestinal expression [46]. Among the upregulated reductases, *Por* encodes NADPH-cytochrome P450 reductase, the essential electron donor for microsomal cytochrome P450 enzymes [49]. Increased *Por* expression may therefore increase the capacity to support cytochrome P450 catalytic activity.

As shown in Table 5, statistically significant over-representation of upregulated genes encoding GSTs, MGSTs and UGTs was observed. UGTs represent a major group of phase II drug-metabolizing enzymes, with a broad range of substrates that includes non-steroidal anti-inflammatory agents. Eight of the 12 UGT genes were upregulated with FDR < 0.01, including all four expressed mouse homologs (*Ugt1a7c*, *Ugt1a8*, *Ugt1a9*, and *Ugt1a10*) that map to the human gene *UGT1A10*, which is highly expressed in both the colon and small intestine [50,51]. Upregulation of genes involved in UDP-glucuronic acid synthesis (Table 6) may increase availability of this required co-substrate and thereby affect rates of UGT-mediated glucuronidation, as endogenous UDP-glucuronic acid concentrations have been shown to constrain glucuronidation in the intestine and liver [52]. The net effect on intestinal xenobiotic disposition, however, may also depend on microbial β-glucuronidase activity, which reverses glucuronidation by hydrolyzing glucuronide conjugates, releasing the parent compound and glucuronic acid [53].

Also upregulated were 12 genes encoding GST/MGST enzymes (Table 5). Butyrate, a major product of gut microbial fermentation, has been shown to increase the expression of several GST isoforms in human colonic cells [54]. Increased expression of glutathione-synthesis genes (Table 6) is less likely to increase GST activity directly because mu-class GSTs, which account for most of the upregulated GSTs, have low K_m_ values for glutathione relative to typical intracellular glutathione concentrations [55]. The upregulation of genes encoding both GST enzymes and glutathione-synthesis enzymes, however, is consistent with activation of the Nrf2/antioxidant response element pathway and thus suggests increased GST capacity [56].

Efflux transport is another major determinant of intestinal drug absorption. P-gp, arguably the most clinically relevant such transporter, showed similar increases in both gene and protein expression. P-gp is a predominant drug efflux transporter that reduces the bioavailability of a wide range of therapeutics—particularly moderately lipophilic, neutral, or weakly basic drugs with limited hydrogen bonding [57,58].

*Abcg2* and *Abcc3*, which encode the efflux transporters BCRP and MRP3, respectively, were also upregulated in humanized colon (Figure 7). The activities of P-gp and BCRP in enterocytes decrease bioavailability, while MRP3 is localized at the basolateral surface and may increase the bioavailability of drug substrates or enzyme-activated prodrugs [59]. MRP3 transports glutathione, sulfate, or glucuronide conjugates of substrates such as etoposide and methotrexate [60], linking its upregulation to the observed increases in phase II enzyme expression.

Among the upregulated solute carriers in Table 7, *Slc22a5* (OCTN2) is notable because its human ortholog is a significant drug transporter in the small intestine. OCTN2 is an apically polarized influx transporter specialized in the absorption of endogenous zwitterion carnitine, a fatty acid shuttle, and a regulator of acyl-CoA balance [61]. In humans, OCTN2 also functions as an important transporter for many other ionic and neutral compounds, including imatinib (anticancer agent), ipratropium (bronchodilator), verapamil (calcium channel blocker), valproic acid (anticonvulsant/mood stabilizer), and quinidine (antiarrhythmic agent) [16,61]. In addition, *Slc16a1*, which encodes MCT1, demonstrated a two-fold upregulation. In humans, intestinal MCT1 is mainly known for transporting metabolic monocarboxylates such as lactate, pyruvate, and ketone bodies [62], but is also known as a transporter of weakly acidic drugs with a carboxylic acid moiety, such as fluorouracil and gemcitabine prodrugs [63,64] (anticancer agents), and γ-hydroxybutyrate [65] (narcolepsy treatment). Although salicylic acid has been suggested as an MCT1 substrate, its involvement in human MCT1 transport remains uncertain [66].

By systematically identifying host genes that respond to microbial colonization, these findings define candidate pathways in mice through which microbiota may influence intestinal drug absorption and disposition. Because the germ-free-to-humanized comparison represents a strong contrast in microbial exposure, it provides a useful first-pass screen and narrows the hypothesis space for evaluating how drug transport and metabolism may differ across human microbiome profiles, including disease-associated microbiome states. Beyond these pharmacokinetic processes, this study provides a reference of microbiota-responsive gene expression in mouse colon that could support future studies of colonic physiology, homeostasis, and disease.

### Limitations

This study has several limitations. All humanized mice were generated using fecal microbiota from a single donor, which cannot account for inter-donor variability or exclude the possibility that some responses were influenced by donor-specific features. Transcriptomic profiling was conducted in colon tissue only. Small intestinal tissue, the principal site of drug absorption, was not profiled, and the relationship between colonic and small intestinal responses to microbiota remains to be tested. Transcriptomic and immunofluorescence profiling was also conducted in male mice only, and this study cannot rule out sex-specific differences in the host response to microbiota. The sample size for P-gp immunofluorescence was small (*n* = 2 germ-free, *n* = 3 humanized), which limits the precision of that estimate. The colonoid efflux assay was qualitative, and interpretation of its results is confounded by esterase-mediated calcein-AM hydrolysis. In addition, the colonoids had the standard apical-in orientation, in which the apical membrane faces an enclosed central lumen and P-gp-mediated transport would be directed into the lumen rather than out of the imaged structure [67,68]. Whole-colonoid fluorescence therefore cannot be interpreted as a measurement of P-gp efflux activity.

Finally, although the discussed mouse genes have functionally expressed human counterparts in the intestine, each gene-level result should be regarded as a distinct hypothesis regarding microbiota-dependent regulation of the corresponding human gene. Whether each human homolog exhibits a similar response depends on complex and incompletely understood microbial, cellular, and regulatory factors. Consequently, conservation of ortholog mapping, gene structure, or known physiological function does not necessarily predict conservation of microbiota-dependent gene regulation.

## 4. Materials and Methods

### 4.1. Mouse Husbandry

Germ-free Swiss Webster mice were obtained from the Mayo Clinic Germ-Free facility, and humanized mice were generated from germ-free Swiss Webster mice. Both germ-free and humanized mice were housed in flexible film vinyl isolators (Class Biologically Clean, Madison, WI, USA) within the Mayo Clinic Germ-Free facility. Mice were singly housed in open-top cages containing autoclaved Sani-Chips bedding, provided with an autoclaved diet (Purina Lab Diet, 5K67, Collins Feed and Seed Center, Rochester, MN, USA) and autoclaved nanopure water ad libitum. Bedding and feed were changed weekly or more frequently as needed. Germ-free status was confirmed prior to experiments by two consecutive cultures of fecal pellets, feed, and bedding on three different substrates: brain heart infusion, Sabouraud dextrose, and nutrient media—all under both anaerobic and aerobic conditions. Mice were maintained on a 12 h light–dark cycle and monitored daily for any signs of physical stress or behavioral changes. Any animal exhibiting distress was euthanized in accordance with the approved protocol. To minimize experimental bias, animal experimenters were provided with information necessary to perform the procedures and were blinded to details of the final data analysis and study conclusions until completion of all animal experiments. All studies were conducted in accordance with the National Institutes of Health guidelines for the care and use of laboratory animals and were approved by the Mayo Clinic Institutional Animal Care and Use Committee. Data are reported in compliance with the ARRIVE guidelines.

Germ-free mice were humanized by oral gavage of fecal microbiota from a single healthy human donor (49-year-old male) at 4–5 weeks of age. A single donor was used to minimize inter-donor variability and maximize statistical power for mechanistic discovery. We have previously published multiple studies demonstrating microbiome composition and engraftment stability following humanization with healthy human stool using the same protocol [69,70,71]. The human fecal sample used in the present study was the same donor sample whose microbiota composition was characterized by Kashyap et al. [71].

The stool suspension was prepared by mixing equal volumes of stool and sterile pre-reduced Phosphate-Buffered Saline (PBS) inside an anaerobic chamber (Coy Laboratory Products, Grass Lake, MI, USA). The sealed vial was then removed from the chamber and transferred to germ-free isolators. Each mouse received 300 μL of the stool suspension via gavage and was allowed 4–5 weeks for microbiota adaptation to the mouse gut. All experiments were conducted when mice were 8–10 weeks of age. Separate, non-overlapping cohorts of germ-free and humanized mice were used for transcriptomic, permeability, and immunofluorescence analyses.

### 4.2. Transcriptomics: Library Preparation, Sequencing, and Alignment

Colonic tissue from germ-free (*n* = 5) and humanized (*n* = 6) mice was used for transcriptomic analyses. All mice were male. Messenger RNA was extracted from mouse samples according to the Illumina TruSeq RNA Library Prep Kit v2 instructions (Illumina, San Diego, CA, USA). Sequencing was performed using an Illumina HiSeq-3000 by the Mayo Clinic Medical Genome Facility (Illumina, San Diego, CA, USA) with 101 bp paired-end reads. Sequencing data were collected with Illumina HCS v3.3.52. Base-calling was performed with Illumina RTA v2.7.3 and converted to FASTQ with Illumina BCL2FASTQ v2.17.1.14. The FASTQ files were processed using the Mayo Clinic in-house pipeline MAP-RSeq v.2.1.1 workflow [72]. Sequences were aligned with TopHat (2.1.0) and Bowtie (2.2.3) against the mouse genome build mm10. Gene counts were quantified using featureCounts from Subread package 1.4.6-p5 with mm10 mouse gene annotation [73]. The resulting counts matrix with 43,346 Mus musculus Ensembl genes is included in the Supplementary Materials.

### 4.3. Transcriptomics: Analysis

A human homolog database was constructed with 26,281 mouse–human homologs, including one-to-many mappings in either direction from the “complete list of human and mouse homologs” downloaded from the Mouse Genome Informatics’ website https://www.informatics.jax.org/homology.shtml (accessed on 24 June 2024). All statements regarding human-mouse homologs in this study are based on these homology assignments. Low-expression filtering was performed using edgeR 4.4.2’s filterByExpr() function with default parameters (min.count = 10, min.total.count = 15, large.n = 10, min.prop = 0.7). Trimmed mean of M-value (TMM) normalization, dispersion estimation, and differential expression testing with Benjamini–Hochberg multiple hypothesis correction were performed with edgeR 4.4.2, using the classic edgeR pipeline with exact tests and default parameters. A total of 19,863 genes remained after low-expression filtering. Relative gene abundance was estimated as TMM-normalized RPKM (Reads Per Kilobase per Million mapped reads) values using edgeR’s rpkm function. Percentile ranks were computed from the distribution of gene abundance values pooled across all samples from both treatment groups. Results of normalization, differential expression, and relative abundance estimation are included in Supplementary Files. Over-representation of differentially expressed genes in phase II enzyme families was assessed using a one-sided Fisher’s exact test. The background universe comprised all 19,863 genes included in the differential expression analysis, and each enzyme-family set included annotated genes with mean RPKM > 4 in either treatment group.

### 4.4. Colonoid Preparation

Three-dimensional colonoids were prepared by isolating the proximal colon segment from 8- to 10-week-old germ-free and humanized mice (sex not recorded). Intestinal crypts were centrifuged after suspension in cold DMEM/F12 medium (Life Technologies, Carlsbad, CA, USA), and the pellet was resuspended in the IntestiCult™ Organoid Growth Medium (StemCell Technologies, Vancouver, BC, Canada) on ice. Reduced Matrigel (1:1 ratio, Corning, Corning, NY, USA) was then added to the crypt suspension and mixed thoroughly. An 80 µL aliquot of this suspension was added to a pre-warmed 24-well plate, creating a dome-like structure, which was incubated at 37 °C for 10 min before adding medium supplemented with penicillin/streptomycin. The medium was changed every 3–4 days, depending on colonoid growth. After 7 days, colonoids were passaged by re-dispersing them with a pipette in gentle Cell Dissociation Reagent (StemCell Technologies, Vancouver, BC, Canada) at 37 °C for 15 min. Dispersed colonoids were diluted with DMEM/F12 and pelleted by centrifuging (290× *g*) for 5 min at 4 °C. The pellet was then resuspended in colonoid growth medium and plated on coverslip-bottom dishes.

### 4.5. Colonoid Fluorescence Imaging

Qualitative imaging studies were conducted in colonoids using calcein-acetoxymethyl ester (calcein-AM), a P-gp substrate that becomes fluorescent following intracellular esterase hydrolysis. In the control group, colonoids grown on coverslips were incubated with calcein-AM (0.5 µM) for 60 min at 37 °C. For inhibition studies, colonoids were pretreated with the P-gp inhibitor cyclosporine A (5 µM) for 30 min at 37 °C prior to addition of calcein-AM. Live images were taken at 37 °C and 5% CO_2_ using a Nikon TE2000-S inverted microscope (Chiyoda-ku, Tokyo, Japan) equipped with a Nikon FITC HQ filter (Nikon, Tokyo, Japan) and a Zeiss Axio Observer Z1 spinning disk laser confocal microscope (Zeiss, Oberkochen, Germany) equipped with a Photometrics QuantEM 512C EMCCD camera (Photometrics, Tucson, AZ, USA). All images were processed in Fiji software (v1.54f) with automated background subtraction using a rolling-ball radius of 50 pixels. Colon tissue was pooled across multiple mice to generate colonoids for this qualitative imaging assay. Animal-level replicate numbers were therefore not preserved. Colonoid images shown are representative; no quantitative image analysis was performed.

### 4.6. Immunofluorescence

Colonic tissue was collected from mice and fixed with 4% paraformaldehyde in 1X Dulbecco’s PBS at 4 °C for 4 h. Tissue was then dehydrated in a gradient of solutions with various concentrations of sucrose [10% (*w*/*v*), 20% (*w*/*v*), and 30% (*w*/*v*)] over 3 days at 4 °C. After dehydration, the tissue was embedded in the Tissue-Tek O.C.T. compound (Sakura Finetek, Torrance, CA, USA). The embedded tissue was sectioned into 8 µm slices using a cryostat (Leica Biosystems CM3050 S Research Cryostat, Leica, Berlin, Germany) and mounted on slides. Endogenous peroxidase activity was blocked by incubating the sections in 3% hydrogen peroxide in distilled water. Subsequently, sections were blocked using goat serum (1:10 in 0.02 M PBS) for 20 min at room temperature. The blocked sections were incubated overnight at 4 °C with a rabbit anti-P-gp antibody (RP1034, Boster Biotechnology, Pleasanton, CA, USA, 1:200). The next day, tissue was washed three times with PBS, followed by incubation with a biotinylated goat anti-rabbit IgG secondary antibody (SABC kit SA1074, Boster Biotechnology, Pleasanton, CA, USA, 1:200) at room temperature for 30 min. Tertiary antibody staining for P-gp was performed using Cy3-conjugated streptavidin (SABC-Cy3, 1:200) at room temperature for 30 min in the dark. Finally, nuclei were stained by incubating the tissue with 4′,6-diamidino-2-phenylindole (DAPI, 1:5000 in 0.02 M PBS). Immunofluorescence was detected using a Leica inverted fluorescence microscope (Leica Microsystems, Buffalo Grove, IL, USA) and captured using Leica Application Suite X (LASX) software v3.1.1. Images from male germ-free (*n* = 2) and male humanized colon samples (*n* = 3) were analyzed using FIJI software (v1.54f). After threshold background removal, the mean intensity of each image sample was calculated. A mean intensity of 50 manually selected points along the apical surface was also calculated. Point-level values were averaged into a single value per animal, and group means were then compared by a two-tailed unpaired Student’s *t*-test (*n* = 2 germ-free, *n* = 3 humanized).

### 4.7. Permeability Studies

Gastrointestinal mucosal permeability of small intestinal tissue from germ-free and humanized mice was assessed in vitro using Ussing chambers (Physiologic Instruments, FL, USA). After small intestine tissue was collected, it was opened along the mesenteric border and washed in ice-cold Krebs’ solution. The mucosa–submucosa epithelial sheet was mounted onto the Ussing chamber cassette such that the serosal side faced up, and the muscle layer was peeled away. The basolateral and apical sides of the chambers contained glucose-containing Krebs solution and Krebs-mannitol solution, respectively. ^14^C-labeled polyethylene glycol 4000 (^14^C-PEG 4000) was added (0.25 µCi) to the mucosal side, and the samples were collected from the serosal side at 1, 5, 10, 15, 30, 60, and 90 min to determine the permeability as described previously [70,74]. Radioactivity in the serosal compartment was quantified using a beta counter. The flux was determined from the slope of the linear portion of the cumulative amount in the serosal compartment versus the time plot, and the permeability was determined by normalizing flux with the initial donor concentration of ^14^C-PEG 4000. Two humanized mouse tissue preparations were excluded according to the prespecified exclusion criterion of chamber leakage. A two-tailed Welch’s *t*-test was used to compare permeability between germ-free (*n* = 6) and humanized mice (*n* = 4). Sex was equally balanced within each group.

## Figures and Tables

**Table 1:** Results of differential expression analysis showing the number of genes corresponding to various thresholds of statistical significance and effect size. Blue denotes genes with FDR < 0.01. Green indicates genes with log2-fold change absolute magnitude of greater than 0.58 (corresponding to fold changes of 2/3 and 3/2). Red indicates genes with both FDR < 0.01 and absolute log2-fold changes greater than 0.58.

		Downregulated (LFC < 0)			Upregulated (LFC > 0)		
	All	<0	<−0.58	<−1	>0	>0.58	>1
All	19,863	9688	2875	1646	10,175	1230	556
FDR < 0.05	5685	2905	2169	1528	2780	897	476
FDR < 0.01	3826	1999	1642	1273	1827	739	405
FDR < 0.001	2365	1263	1084	888	1102	594	320

**Table 2:** Differential expression and relative abundance of genes encoding tight junction-related proteins in mouse colon. LFC is the log2-fold change comparing humanized and germ-free mouse colon. RPKM represents TMM-normalized mean RPKM values in the humanized samples, and “%” represents the percentile in the humanized RPKM distribution. All claudin, occludin, zonula occludens, and myosin II-related genes with RPKM > 4 are included. Genes with FDR < 0.01 are shown in bold.

Gene	LFC	FDR	RPKM	%
Tight Junctions				
***Cldn23***	**1.11**	**2.8 × 10^−23^**	**50**	**87**
***Cldn4***	**1.21**	**2.8 × 10^−11^**	**34**	**79**
***Tjp3***	**0.32**	**5.6 × 10^−3^**	**80**	**96**
*Tjp2*	0.14	2.3 × 10^−1^	26	87
*Cldn3*	0.18	3.6 × 10^−1^	333	100
*Cldn12*	−0.10	3.9 × 10^−1^	18	83
*Cldn15*	0.11	4.3 × 10^−1^	109	98
*Cldn2*	−0.26	4.4 × 10^−1^	79	98
*Tjp1*	−0.11	5.0 × 10^−1^	20	86
*Cldn7*	0.13	5.1 × 10^−1^	309	100
*Cldn25*	0.09	5.6 × 10^−1^	15	79
*Ocln*	0.09	6.3 × 10^−1^	33	90
Myosin				
***Mylk***	**−0.90**	**2.3 × 10^−6^**	**16**	**80**
***Myl9***	**−2.07**	**2.9 × 10^−4^**	**29**	**89**
***Myh11***	**−2.03**	**5.2 × 10^−5^**	**13**	**76**
*Myh9*	−0.01	9.2 × 10^−1^	32	91

**Table 3:** Differential expression and relative abundance of aquaporin water transporter genes. Genes with FDR < 0.01 are shown in bold.

Gene	LFC	FDR	RPKM	%
***Aqp8***	**8.56**	**1.5 × 10^−51^**	**127**	**99**
***Aqp4***	**1.72**	**1.4 × 10^−13^**	**9**	**69**
***Aqp1***	**−0.48**	**1.4 × 10^−4^**	**63**	**96**
*Aqp11*	0.21	1.1 × 10^−1^	21	85

**Table 4:** Non-cytochrome-P450 phase I enzyme genes with human homologs and mean RPKM > 4. For carboxylesterase and carbonyl reductase families containing differentially expressed genes, all isoforms meeting the expression threshold are shown. Genes in bold are differentially expressed with FDR < 0.01.

Gene	Homolog	LFC	FDR	RPKM	%
Carboxylesterase 2					
***Ces2a***	***CES2***	**4.58**	**1.3 × 10^−183^**	**137**	**58**
***Ces2b***	***CES2***	**2.67**	**1.1 × 10^−41^**	**26**	**50**
***Ces2c***	***CES2***	**1.24**	**1.4 × 10^−23^**	**248**	**98**
***Ces2e***	***CES2***	**1.15**	**1.2 × 10^−7^**	**189**	**97**
Carbonyl reductase					
***Cbr3***	***CBR3***	**2.75**	**1.1 × 10^−55^**	**32**	**54**
***Cbr1***	***CBR1***	**1.05**	**3.4 × 10^−30^**	**169**	**97**
*Cbr4*	*CBR4*	0.16	3.4 × 10^−1^	6	56
Carboxylesterase 1					
***Ces1g***	***CES1***	**1.14**	**1.8 × 10^−6^**	**93**	**93**
*Ces1d*	*CES1*	−0.47	1.9 × 10^−2^	43	95
Other Phase I					
***Akr1b8***	***AKR1B10/15***	**2.02**	**3.5 × 10^−19^**	**25**	**60**
***Nqo1***	***NQO1***	**0.73**	**1.0 × 10^−10^**	**39**	**87**
***Por***	***POR***	**0.51**	**9.0 × 10^−7^**	**45**	**90**

**Table 5:** Over-representation analysis for major phase II drug-metabolizing enzyme groups. Each enzyme-family set included annotated genes with mean RPKM > 4 in either treatment group. Upregulated genes encoding UGTs, GSTs, and MGSTs were over-represented relative to all 19,863 genes included in the differential expression analysis, as determined by a one-sided Fisher’s exact test. Reported *p*-values were Bonferroni-adjusted across the 10 tests.

Process	Genes	Total	Up	Padj (up)	Down	Padj (dn)
glucuronidation	UGTs	12	8	8.6 × 10^−3^	0	1.00
glutathione conj.	GSTs, MGSTs	18	12	4.0 × 10^−4^	0	1.00
sulfonation	SULTs	5	2	0.29	0	1.00
acetylation	NATs	2	0	1.00	0	1.00
methylation	methyltransferases	3	0	1.00	0	1.00

**Table 6:** Differential expression results for genes encoding enzymes that synthesize co-substrates of UDP–glucuronosyltransferases and glutathione S-transferases: UDP-glucuronic acid and glutathione, respectively. All genes show statistically significant increases between approximately 30% and 40% (LFC = 0.40–0.49).

Gene	Enzyme	Final Co-Substrate	LFC	FDR
*Ugp2*	UDP-glucose pyrophosphorylase	UDP-glucuronic acid	0.43	1.0 × 10^−4^
*Ugdh*	UDP-glucose 6-dehydrogenase	UDP-glucuronic acid	0.40	7.7 × 10^−3^
*Gclc*	γ-glutamylcysteine synthetase	glutathione	0.41	5.0 × 10^−4^
*Gclm*	γ-glutamylcysteine synthetase	glutathione	0.49	1.7 × 10^−6^
*Gss*	glutathione synthetase	glutathione	0.46	1.7 × 10^−5^

**Table 7:** Differentially expressed solute carriers with known human homologs and drug substrates filtered by FDR < 0.01. Normalized RPKM is given for humanized mice.

Gene	LFC	FDR	RPKM	Endogenous	Drug Substrates [22,23,24]
*Slc16a1* (MCT1)	1.00	1.5 × 10^−10^	57	monocarboxylates (lactate, pyruvate, ketone bodies, etc.)	monocarboxylate-conjugated anticancer prodrugs, γ-hydroxybutyrate, salicylic acid
*Slc5a8* (SMCT1)	0.82	3.0 × 10^−6^	63	SCFAs, nicotinate	monocarboxylate drugs (5-aminosalicylate, salicylate, benzoate, dichloroacetate, γ-hydroxybutyrate)
*Slc15a1* (PEPT1)	−1.50	4.3 × 10^−7^	4	dipeptides, tripeptides	β-lactam antibiotics, valacyclovir, ACE inhibitor prodrugs
*Slc22a5* (OCTN2)	0.39	4.7 × 10^−4^	27	organic cations/zwitterions	valproic acid, verapamil, cephaloridine, quinidine, ipratropium, imatinib
*Slc10a2* (ASBT)	−0.67	7.1 × 10^−4^	130	bile acids	bile acid-conjugated prodrugs: acyclovir, chlorambucil derivatives
*Slc31a1* (CTR1)	0.43	9.6 × 10^−4^	30	cuprous ions	platinum anticancer drugs (cisplatin, carboplatin, oxaliplatin)

## Data Availability

The transcriptomic data that support the findings of this study are openly available in the NCBI Gene Expression Omnibus (GEO) under accession number GSE317716. All other data presented in this study are contained within the manuscript and its Supplementary Materials.

## Supplementary Materials

The following supporting information can be downloaded at: https://www.mdpi.com/article/10.3390/ijms27177583/s1.

## Abbreviations

The following abbreviations are used in this manuscript:

ABC	ATP-binding cassette
BCRP	Breast Cancer Resistance Protein
Calcein-AM	calcein-acetoxymethyl ester
ECM	extracellular matrix
GST	glutathione S-transferases
MCT1	monocarboxylate transporter 1
MGST	microsomal glutathione S-transferases
MRP3	multidrug resistance-associated protein 3
NOS	nitric oxide synthase
OCTN2	organic cation transporter novel family member 2
PCA	principal component analysis
PEG	polyethylene glycol
P-gp	P-glycoprotein
PIGR	polymeric immunoglobulin receptor
POR	NADPH-cytochrome P450 reductase
ROS	reactive oxygen species
RPKM	reads per kilobase per million mapped reads
TMM	Trimmed mean of M-values
UGT	UDP-glucuronosyltransferases
